# Otolith Strontium Isotope (^87^Sr/^86^Sr) Reveals Mixed Life Histories of *Coilia brachygnathus* in the Middle–Lower Yangtze River Floodplain

**DOI:** 10.3390/ani15233434

**Published:** 2025-11-28

**Authors:** Zhongya Xuan, Yinping Wang, Sheng Wang, Yanping Yang, Chongrui Wang, Silei Liu, Kai Liu

**Affiliations:** 1 Wuxi Fisheries College, Nanjing Agricultural University, Wuxi 214081, China; 2017213002@njau.edu.cn (Z.X.); wangyp@ffrc.cn (Y.W.); 2 Key Laboratory of Freshwater Fisheries and Germplasm Resources Utilization, Ministry of Agriculture and Rural Affairs, Freshwater Fisheries Research Center, Chinese Academy of Fishery Sciences, Wuxi 214081, China; yangyp@ffrc.cn (Y.Y.); liusilei@ffrc.cn (S.L.); 3 Aquatic Conservation and Rescue Center of Jiangxi Province, Nanchang 330096, China; wangsheng_86@163.com; 4 Hunan Fisheries Research Institute and Aquatic Products Seed Stock Station, Changsha 410153, China

**Keywords:** life-history plasticity, life-history diversity, proactive habitat selection, *Coilia brachygnathus*, otolith, ^87^Sr/^86^Sr, partial migration, river-lake connectivity

## Abstract

Life-history plasticity allows fishes to exploit heterogeneous freshwater networks, and proactive habitat selection enhances their capacity to cope with climatic and human-induced stressors. *Coilia brachygnathus*, long regarded as a freshwater resident, nevertheless has a poorly understood habitat use and selection. We reconstructed habitat use of *C. brachygnathus* across the middle-lower Yangtze by coupling water ^87^Sr/^86^Sr baselines with otolith ^87^Sr/^86^Sr transects. We used 60 water samples, compiled published datasets, and measured additional water samples from the mainstem, major tributaries, and connected lakes. In situ ^87^Sr/^86^Sr profiles were smoothed with generalized additive models, revealing significant within-profile variation for all fish. Two modes emerged: (i) residents, whose otolith values remained within local water ranges, consistent with limited movement; and (ii) migrants, whose profiles departed from local baselines, indicating exchanges among lakes, tributaries, and the mainstem. Several fish captured at Anqing and Zhenjiang possess Poyang-like core signatures that declined to mainstem values, while bidirectional shifts were common in the Dongting-Xiang system. These results overturn the residency paradigm, demonstrate flexible partial migration, and indicate proactive habitat selection in the face of environmental change, thereby supporting management that maintains river–lake connectivity and quantifies lake contributions to mainstem stocks.

## Simple Summary

Fish within the same species do not all live the same way; they often exhibit life-history plasticity and ecological agency in their habitat use, to find favorable places or avoid harsh conditions. We studied *Coilia brachygnathus* in the Middle–Lower Yangtze River. To see where fish had been, we used “natural fingerprints” left by the water in tiny ear stones that grow in layers inside each fish. These fingerprints come from strontium, a harmless element that varies from place to place and is recorded as the fish grows. Each part of the basin has a slightly different natural balance between two forms of strontium (two isotopes, often written as ^87^Sr and ^86^Sr). As ear stones grow, the ^87^Sr/^86^Sr fingerprints lock in that local balance, layer by layer, without later change—much like tree rings keep a record of past seasons. We first mapped these fingerprints in the river, its tributaries, and connected lakes and then read the same signals along the growth lines of each ear stone. We found two clear patterns: some fish stayed within one area, while others moved between lakes, tributaries, and the main river. One young fish even appeared to have drifted downstream soon after hatching. These results show that this species is flexible and can change habitats when conditions worsen, such as during extreme drought. Protecting the natural connections between rivers and lakes will help these fish populations persist.

## 1. Introduction

During development and growth, fish often exhibit life-history plasticity and ecological agency (the capacity of fish to actively adjust their behavior and habitat use in response to environmental variability, rather than reacting passively) in their habitat use, adapting flexibly to heterogeneous environments [1,2,3,4]. An increasing body of work emphasizes that, through active habitat choice, resource use, and behavioral adjustment, developing individuals can in turn shape the ecological settings they occupy, rather than merely reacting passively to environmental change [5,6]. In this view, life history is a dynamic reciprocal process between organisms and their environments. This agency enables animals to avoid unfavorable conditions during extreme climatic events or anthropogenic habitat deterioration by relocating to more suitable habitats. Such heterogeneous plastic responses produce divergent life histories within and among populations [3,7,8]. This diversity creates a portfolio effect that buffers environmental, climatic, and anthropogenic disturbances, enhancing resistance and recovery and ultimately reducing extinction risk at local to regional scales [1,4,9].

Understanding life-history diversity and how fish adaptively navigate habitats through behavioral decisions and ontogenetic transitions are fundamental for assessing resource status, elucidating population dynamics, and developing effective conservation and management strategies [10,11]. These insights underpin resource assessment, population dynamics, and evidence-based conservation planning [11,12]. The identification of spawning grounds, protection of nursery areas, and maintenance of migration corridors all depend on an accurate understanding of target species’ life-history traits [13]. It is thus of critical ecological and applied importance, especially in the heavily human-impacted Yangtze River basin.

Among the diverse fish fauna of the Yangtze River, the tapertail anchovy (*Coilia brachygnathus*) is generally considered a freshwater-resident ecotype, in contrast to its anadromous congeners [14,15,16,17,18]. Despite its prevalence in lakes and the Middle–Lower Yangtze River (MLY) mainstem, it remains unclear whether *C. brachygnathus* responds passively to the freshwater environment or actively optimizes its ontogeny through habitat choices [19,20,21]. Owing to methodological constraints, fine-scale details of the species’ life history remain unclear. In 2022, the Yangtze Basin experienced an exceptional heat-drought event during the typical flood season. Poyang Lake suffered its most severe extreme drought in nearly seven decades, resulting in a contracted and desiccated main basin and widespread mortality of benthic invertebrates and fishes [22]. The life-history strategies of aquatic species in the aftermath of such extreme disturbances are of considerable ecological interest. Investigating how *C. brachygnathus* responded to these challenging conditions can provide insights into its behavioral plasticity and resilience. However, the otolith Sr/Ca ratios face fundamental challenges when used to resolve habitat transitions in freshwater settings.

To understand these challenges, it is necessary to consider the principles and limitations of otolith microchemistry. Otoliths are metabolically inert structures in the inner ear of teleost fishes, mineralizing sequentially over time. Their elemental composition can reflect the ambient aquatic environment, providing insights into habitat use [23,24,25]. For instance, significant inflections in otolith Sr/Ca or Ba/Ca ratios can effectively indicate major transitions, such as the shift from freshwater to marine habitats [26]. However, otolith elemental composition is not a direct proxy for water chemistry, as elements must pass through physiological barriers before incorporation [27,28]. Both environmental conditions and physiological processes can influence elemental uptake and deposition. In fresh water, otolith Sr/Ca ratios often display only subtle fluctuations, which are typically weak in magnitude and difficult to interpret [29]. These minor chemical signals are difficult to interpret and can be confounded by individual physiological factors, making it challenging to determine whether the observed variations result from actual habitat transitions or merely reflect physiological noise [28]. This technical bottleneck has severely constrained our understanding of habitat transitions in *C. brachygnathus*. Consequently, the freshwater-resident hypothesis lacks direct evidence, and it remains uncertain whether this species undertakes seasonal or staged movements between connected lakes and the river mainstem.

In contrast to Sr/Ca ratios, the strontium isotope ratio (^87^Sr/^86^Sr) undergoes negligible fractionation during incorporation from water into the otolith, thereby closely reflecting the ambient water ^87^Sr/^86^Sr ratio and providing a robust geochemical fingerprint of freshwater habitats [1,30,31]. The ^87^Sr/^86^Sr ratio in freshwater systems is primarily determined by the age and composition of the underlying bedrock, varying with the weathering and mixing of different catchment end members, and remains stable over ecologically relevant timescales [32,33]. Therefore, otolith ^87^Sr/^86^Sr ratios are particularly well-suited for tracing fish origin and movement among freshwater bodies with distinct isotopic signatures [34,35,36].

The primary goal of this study is to use ^87^Sr/^86^Sr ratios in otoliths to infer habitat use patterns and reconstruct the life-history movement trajectories of *C. brachygnathus* that collected from different water systems (e.g., Dongting Lake, Poyang Lake, Shijiu Lake, and the mainstem). We hypothesize that there exists previously unrecognized staged movement behavior between freshwater systems (e.g., connected lakes and the mainstem) within the *C. brachygnathus* population. The findings will fill a critical knowledge gap regarding this species’ life history, enhance our understanding of ecological divergence mechanisms in closely related species, and provide a scientific basis for assessing river-lake connectivity, habitat protection, and fisheries management. Ultimately, this research will yield key evidence for determining whether *C. brachygnathus* actively optimizes its survival strategy through behavioral plasticity during development.

## 2. Materials and Methods

To construct a baseline of ^87^Sr/^86^Sr ratios for the major potential habitats of *C. brachygnathus*, we integrated existing ^87^Sr/^86^Sr data from the MLY mainstem, its major river-connected lakes (Poyang Lake and Dongting Lake), and the Han River (Table A1). The published bioavailable Sr isotope data were collected from previous studies [37,38,39,40,41]. To ensure comparability across datasets, all ^87^Sr/^86^Sr ratios were normalized to the SRM 987 reference value of 0.710250 using a linear correction method based on the SRM 987 values measured within respective analytical batches. For published datasets in which standard values were not reported, the ^87^Sr/^86^Sr ratios were used directly without further correction.

**Figure 1:**
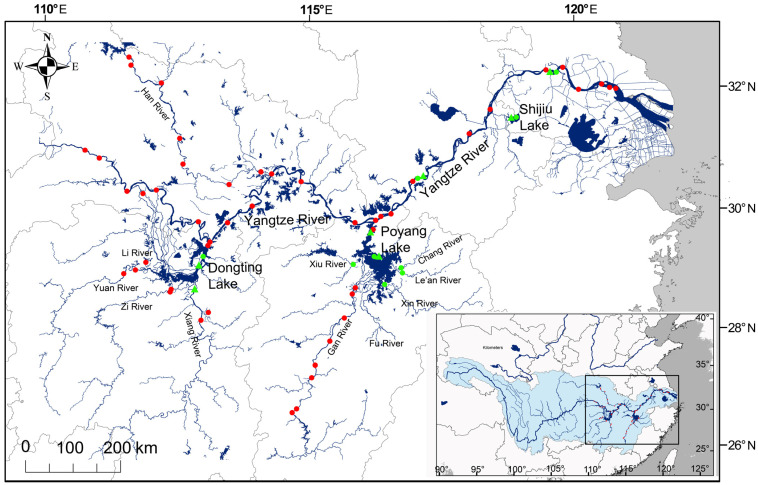
Map of the Middle–Lower Yangtze River showing sampling locations. Red filled circles indicate ^87^Sr/^86^Sr baseline water sampling sites literature-derived; green filled circles indicate water samples collected in this study; green triangles indicate fish sampling sites.

Additionally, water samples were collected from the MLY mainstem and the major tributaries of three river-connected lakes in August 2023 and January–April 2024 (Figure 1 and Table A1). This sampling strategy targeted regions with high migration potential for *C. brachygnathus*. The blocked lakes and smaller tributaries, where migration was restricted by dams and sluices, were not sampled. Water samples were filtered in situ, stored in acid-washed 250 mL polypropylene bottles, refrigerated at 4 °C, and processed within 48 h. All samples contained sufficient Sr for reliable ^87^Sr/^86^Sr ratio determination. In a clean laboratory, the samples were further filtered, acidified with nitric acid (Suprapur^®^ grade, Merck KGaA, Darmstadt, Germany), and evaporated, and Sr was purified using Sr-specific resin (SR-B200-A, Triskem International, Bruz, France). The ^87^Sr/^86^Sr ratios were measured by multi-collector inductively coupled plasma mass spectrometry (MC-ICP-MS) (Neptune Plus, Thermo Fisher Scientific, Bremen, Germany). Repeated measurements of the Sr isotope standard reference material SRM 987 were performed to assess the accuracy and reproducibility of the entire procedure. The measured ^87^Sr/^86^Sr ratio for NIST SRM987 was 0.710247 ± 0.000017 (2SD), consistent with the reported value. Sample ^87^Sr/^86^Sr ratios were normalized relative to the concurrently measured NIST SRM 987 value.

Fish specimens were collected from Duchang and Hukou in Poyang Lake, Yueyang in Dongting Lake, Xiangyin in the Xiang River, Shijiu Lake, and the Anqing and Zhenjiang sections of the Yangtze mainstem between June and July 2023, with permission from the local Department of Agriculture and Rural Affairs (Figure 1). A standardized sampling methodology was employed across all localities to ensure comparability. All fish were captured using monofilament gill nets with a uniform stretched mesh size of 40 mm. Specimens were identified as *C. brachygnathus* based on morphological characteristics, specifically a maxilla-to-head length ratio < 1 [17]. The estimates of sexual maturity in *C. brachygnathus* differ slightly between sexes. The length at 50% maturity (L50) is 17.2 cm for females and 19.0 cm for males, corresponding to approximately 1 year and 1.6 years of age, respectively [42]. In the present study, all analyzed individuals exceeded 25 cm in total length. Therefore, the majority, if not all, of the specimens were likely reproductively mature adults, and their otolith profiles are considered to represent typical life histories [42]. Collected samples were labeled, placed in plastic zip-lock bags, refrigerated, promptly transported to the laboratory, and stored frozen. Basic biological information was recorded for each specimen, including the total length (TL, to the nearest 1 mm), standard length (SL, to the nearest 1 mm), and body weight (to the nearest 0.01 g) (Table 1).

Sagittal otoliths were extracted from the fish heads and cleaned of adhering tissue. To achieve high spatial resolution, sections were cut through the sagittal plane to intersect the longest growth axis. The otoliths were embedded in epoxy resin. After hardening, the sagittal plane was ground sequentially using 1200, 2400, and 4000 grit waterproof sandpaper (Struers, Ballerup, Denmark) until the core was exposed, followed by polishing with a silica suspension on a polishing cloth. Polished otoliths were ultrasonically cleaned for 5 min, rinsed with deionized water, air-dried, and stored for subsequent analysis [14].

In situ ^87^Sr/^86^Sr ratios in otoliths were obtained using laser ablation multi-collector inductively coupled plasma mass spectrometry (LA-MC-ICP-MS) (laser ablation: RESOlution SE 193-nm laser ablation system, Applied Spectra, Fremont, CA, USA; MC-ICP-MS: Neptune Plus). Laser parameters included an energy density of ~6 J/cm^2^, a spot size of 50 µm, a repetition rate of 6 Hz, and a scan speed of 10 µm/s. Line transects were ablated from the core to the edge of each otolith. Background intensities for all isotopes were measured for 20 s prior to each ablation and averaged for blank correction. The otolith of the marine fish Eleutheronema tetradactylum was used as a reference material for the ^87^Sr/^86^Sr value of marine calcium carbonate and was analyzed every five unknowns. Data reduction and correction followed the method described by Lugli et al. [43]. Krypton interferences were corrected using the on-peak zero method. Potential ^40^Ar^42^Ca interference was monitored at *m*/*z* 82, but signal intensities were consistently below 0.0003, indicating negligible interference. Although the measured ^85^Rb signals were consistently less than 0.003% of ^88^Sr, ^87^Rb corrections were still applied to ensure analytical rigor. The ^85^Rb signal was used to correct for ^87^Rb interference at *m*/*z* 87, with the mass bias adjusted using an exponential law. Final ^87^Sr/^86^Sr ratios were then corrected for mass bias accordingly. Isotope data were acquired in low-resolution static mode. The measured ^87^Sr/^86^Sr ratio for the marine calcium carbonate reference material (0.70918 ± 0.00008, 2SD) during the analytical sessions matched the modern global seawater value of 0.70918. After ablation, the otoliths were etched with 5% EDTA following established methods and photographed under a microscope, and the annuli were counted under reflected light following Xuan et al. [14].

The ^87^Sr/^86^Sr ratios from the public database and those obtained in this study were used to establish the natal ^87^Sr/^86^Sr baseline. Prior to analysis, the normality of distribution and homogeneity of variances for the ^87^Sr/^86^Sr data across regions were tested. The results indicated that the ^87^Sr/^86^Sr ratios did not follow a normal distribution (Shapiro–Wilk test, *p* < 0.001), and significant heteroscedasticity was observed (Levene’s test, *p* < 0.05). As data transformation failed to meet these assumptions, differences in ^87^Sr/^86^Sr ratios among regions were assessed using the Kruskal–Wallis test, followed by Dunn’s post hoc pairwise multiple comparisons. The Benjamini–Hochberg method was used to adjust *p*-values for multiple comparisons. The significance level for all tests was set at α = 0.05.

The average ^87^Sr/^86^Sr ratio from the outermost 50 µm of each otolith transect was calculated, representing a natural tag of the recent environment prior to capture. Similarly, the average ^87^Sr/^86^Sr ratio from the innermost 50 µm near the otolith core was used as a marker for the natal period.

To smooth the variation in ^87^Sr/^86^Sr ratios from the core to the edge of adult *C. brachygnathus* otoliths, we applied a generalized additive model (GAM) using the mgcv package (Version 1.9) in R (Version 4.1) flowing the approach described by a previous study [44]. The model structure was defined as: ^87^Sr/^86^Sr ~ s(Distance_from_Core), where ‘Distance_from_Core’ represents the sequential sampling points along the laser transect. The GAM-fitted ^87^Sr/^86^Sr profiles, along with their Bayesian 95% confidence intervals, were visualized. To guard against prematurely over-penalizing any GAM, the basis dimension (*k*), also known as the maximum allowable effective degrees of freedom (edf), was defined as *k* = 10*N*^2/9^, where *N* represents the number of laser sampling points per otolith transect (varying for each otolith) [44]. This formula scales the complexity ceiling (*k*) with the length of the time series (*N*), providing a consistent and data-driven approach for all specimens. Comprehensive residual diagnostics were performed for each fitted GAM to validate the model assumptions and goodness-of-fit. This included the examination of three primary diagnostic plots: (1) a Quantile-Quantile (Q-Q) plot to assess the normality of the residuals; (2) a plot of residuals against the linear predictor to detect any systematic biases or heteroscedasticity; and (3) a plot of response values against fitted values to evaluate how well the model captured the major trends in the data.

## 3. Results

### 3.1. Water ^87^Sr/^86^Sr Baselines

**Figure 2:**
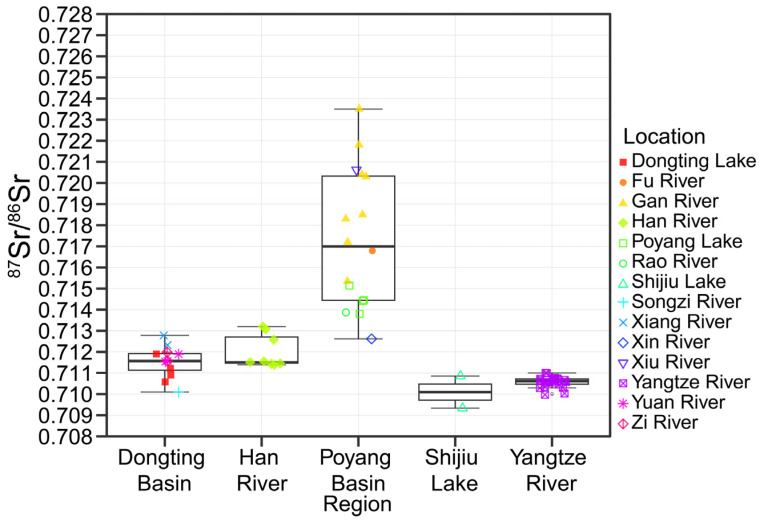
Boxplot showing the ^87^Sr/^86^Sr ratios of the Middle–Lower Yangtze River, Han River, Poyang Lake Basin, Dongting Lake Basin, and Shijiu Lake. The boxes represent the inter quantile range calculated as Q3-Q1; lines = mean; whiskers = maximum and minimum values. Different shapes represent the water samples.

We collected 60 water samples from various locations to characterize the geochemical signatures of the main channel, major tributaries, and connected lakes in the MLY (Figure 2). Significant spatial heterogeneity in ^87^Sr/^86^Sr ratios was observed across different geological regions (large geographic units that include lake areas and tributaries) (Kruskal–Wallis test, *p* < 0.001). Poyang Lake exhibited the highest ^87^Sr/^86^Sr ratios, which were significantly distinct from those in the MLY mainstem (Dunn’s test, *p* < 0.001), Han River (Dunn’s test, *p* < 0.05), Dongting Lake (Dunn’s test, *p* < 0.01), and Shijiu Lake (Dunn’s test, *p* < 0.05). The ^87^Sr/^86^Sr ratios of the MLY mainstem also differed significantly from those in Dongting Lake (Dunn’s test, *p* < 0.05) and the Han River (Dunn’s test, *p* < 0.01). In contrast, no significant differences were observed between Shijiu Lake and the other regions (Dunn’s test, *p* > 0.05). Within the Poyang Lake system, the five major tributaries and the lake itself exhibited ^87^Sr/^86^Sr ratios consistently higher than 0.713, exceeding the values recorded in other regions, with an exception of the Xin River (Figure 2).

When evaluated separately by hydrological unit (mainstem, lakes, and tributaries of lakes), the ^87^Sr/^86^Sr baselines of Poyang Lake displayed the higher ^87^Sr/^86^Sr baselines (0.71390–0.71499), which was isotopically unique and did not overlap with any other region. The tributary of Poyang Lake, Gan River, displayed 0.71681–0.72203. In contrast, Dongting Lake, the Yangtze River, and Han River showed substantial overlaps in their isotopic ranges, and overlaying shaded bands representing these regions on the otolith profiles would create visual confusion rather than enhance interpretation. Therefore, we present the complete baseline statistics in Table 2 to provide accurate quantitative reference for all regions (Table 2).

### 3.2. Otolith Edge ^87^Sr/^86^Sr and Recent Habitat Use

**Figure 3:**
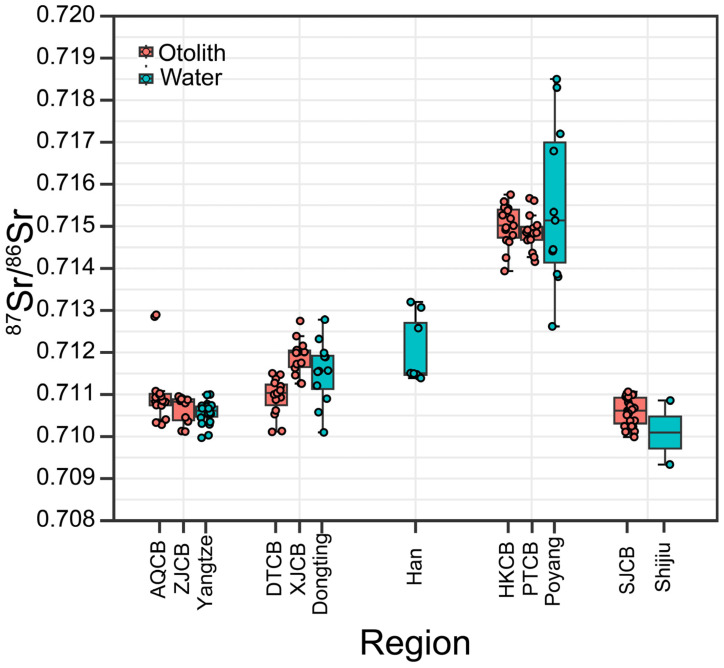
Boxplot showing the otolith and water ^87^Sr/^86^Sr ratios of different regions. The boxes represent the inter-quantile range calculated as Q3-Q1; lines = mean; whiskers = maximum and minimum values of a region. Spots representing the isotopic ratio of single sample. Light red boxplot and spot represent otolith edge ^87^Sr/^86^Sr ratios, light blue boxplot and spot represent water ^87^Sr/^86^Sr ratios.

The ^87^Sr/^86^Sr ratios measured at the edge of *C. brachygnathus* otoliths generally aligned with the baseline values of their capture locations, indicating residence in those habitats prior to capture. However, exceptions were noted: four individuals from Shijiu Lake and two from Anqing exhibited edge ^87^Sr/^86^Sr ratios that fell outside the range of their respective local water samples (Figure 3).

### 3.3. Life-History Patterns Revealed by Otolith Transects

**Figure 4:**
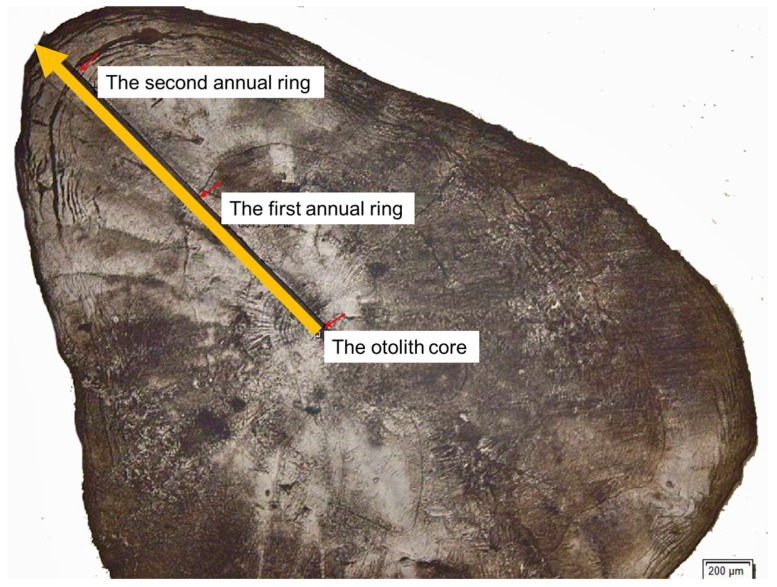
Laser-ablated paths from core to edge on otoliths of adult *Coilia brachygnathus*. Yellow lines and arrows indicate the direction and path of laser ablation, and red arrows mark the location of otolith core and growth rings.

Line transect analyses using LA-MC-ICP-MS captured fine-scale variations in the ^87^Sr/^86^Sr ratios across the otoliths of *C. brachygnathus* individuals. The positions of annual growth rings (annuli) were identified for each otolith and annotated along the corresponding ^87^Sr/^86^Sr profiles. An example showing the annuli is presented in Figure 4.

The GAMs successfully captured the significant non-linear variation along all ^87^Sr/^86^Sr profiles, including profiles that appeared visually flat yet exhibited significant smooth terms. The smooth term for ‘Distance_from_Core’ was statistically significant for every specimen (all *p*-values < 0.01), with the edf ranging from 3.02 to 48.17, confirming that none of the profiles were simple linear trends.

Residual diagnostics confirmed the robustness of the GAM fits. The Q-Q plots showed that the residuals for all individuals fell approximately along the diagonal line, indicating a distribution close to normality. The plots of residuals versus the linear predictor showed no discernible systematic pattern or funnel shape, suggesting homoscedastic variance and no major model bias. Furthermore, the plots of response versus fitted values demonstrated a strong agreement, with points distributed tightly around the 1:1 line, confirming that the models effectively captured the underlying trends in the ^87^Sr/^86^Sr data.

Based on these profiles, two predominant life-history modes were identified: (1) resident individuals, whose entire ^87^Sr/^86^Sr profiles remained within the ^87^Sr/^86^Sr range of their capture location’s water baseline, and (2) migratory individuals, exhibiting ^87^Sr/^86^Sr signatures indicative of movement between isotopically distinct water bodies.

**Figure 5:**
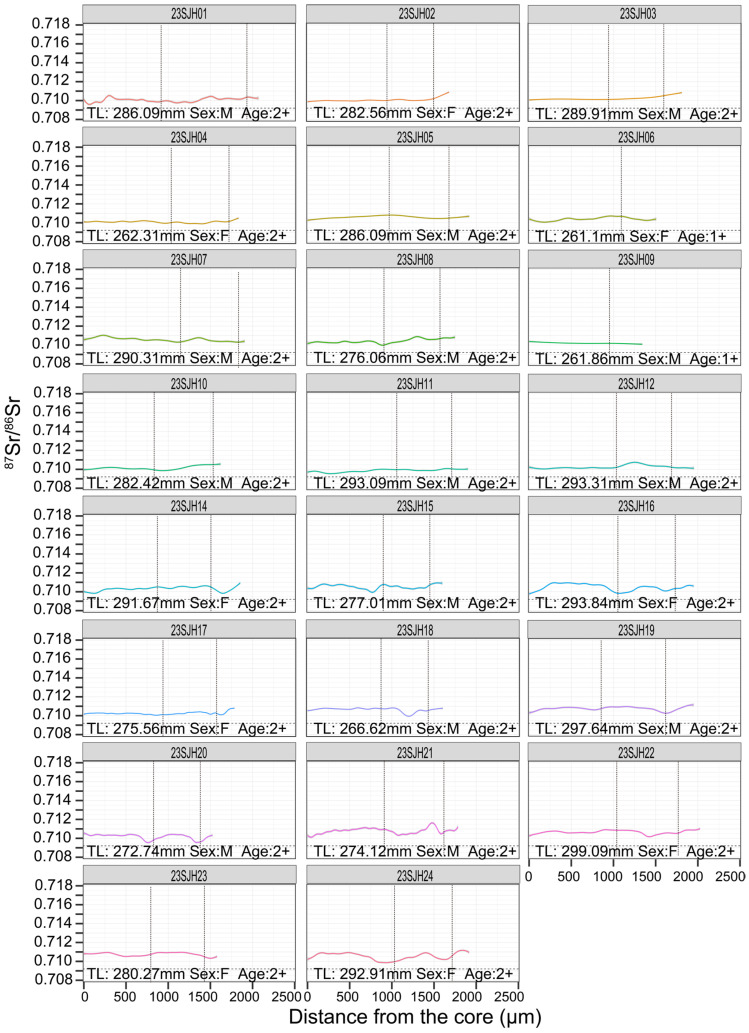
Otolith ^87^Sr/^86^Sr profiles of *Coilia brachygnathus* from Shijiu Lake, 2023. The position of the vertical dashed lines represents the annuli.

In the Shijiu Lake population, most individuals exhibited limited temporal variance in ^87^Sr/^86^Sr profiles. Several fish (23SJH08, 14, 15, 16, 18, 19, 20, 21, 22, 23, 24) exhibited one or two brief declines, yet all shifts remained consistent with the local winter ^87^Sr/^86^Sr ratio. (Figure 5). These were classified as local residents.

**Figure 6:**
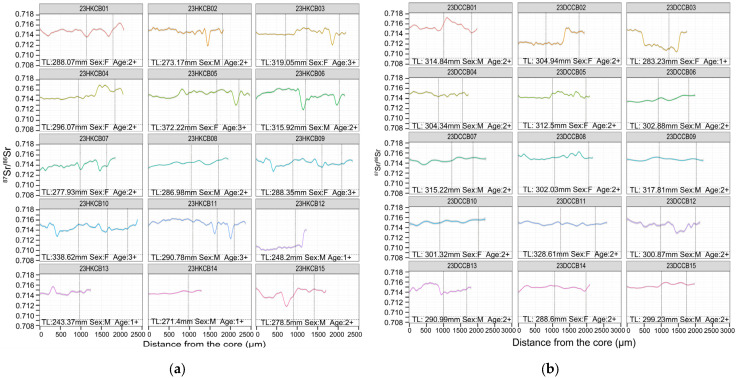
Otolith ^87^Sr/^86^Sr profiles of *Coilia brachygnathus* from Poyang Lake, 2023. The position of the vertical dashed lines represents the annuli. (**a**) The specimen from Hukou reach of Poyang Lake; (**b**) the specimen from Duchang reach of Poyang Lake.

For the population from Poyang Lake, Hukou reach, individuals 23HKCB04, 08, 13, and 14 remained primarily above 0.714. Fish 23HKCB01, 05, 07, 09, and 10 displayed one or two modest short-lived decreases. Individuals 23HKCB02, 03, 6, 11, and 15 each showed a single decline that fell below 0.713. Only 23HKCB12 exhibited a prolonged early-life segment between the core and ~1100 µm at 0.710–0.711, followed by a rise above 0.713 near the edge, consistent with cross-regional movement (from MLY mainstem to Poyang Lake) and thus assigned to the migratory mode (Figure 6a). For the population from Poyang Lake, Duchang reach, most individuals tracked the lake baseline with minimal variation. Several fish showed pronounced deviations: 23DCCB02 began near 0.712—close to the Xin River value—then rose above 0.714, consistent with cross-regional movement (from Xin River to Poyang Lake) and was thus assigned to the migratory mode. In contrast, 23DCCB03 started near 0.714, declined to ~0.712 around 500 µm, held a short plateau, then dropped further to 0.710–0.711 before rising again to ~0.714 near the edge. Individual 23DCCB12 declined to ~0.713 for an interval and later returned to ~0.714 (Figure 6b). These profiles suggest cross-basin movements.

**Figure 7:**
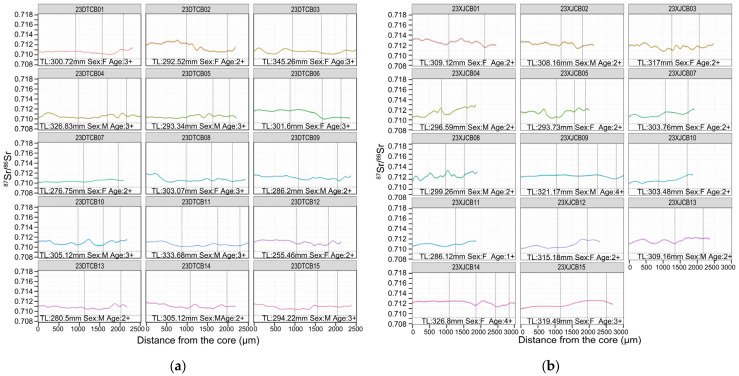
Otolith ^87^Sr/^86^Sr profiles of *Coilia brachygnathus* from Dongting Lake, 2023. The position of the vertical dashed lines represents the annuli. (**a**) The specimen from Yueyang reach of Dongting Lake; (**b**) the specimen from Xiang River reach of Dongting Lake.

For Dongting Lake, Yueyang reach, individuals 23DTCB02, 06, 08, 14, and 15 started near 0.712 and subsequently declined toward ~0.710, indicating movements between the Xiang River and the lake. Other Yueyang individuals remained mostly around 0.710, with occasional brief upward excursions (Figure 7a). For the Xiang River reach, individuals 23XJCB04, 10–13, and 15 began near ~0.710 and then increased above 0.712, consistent with movement between the Xiang River and Dongting Lake. Other Xiang River fish remained above 0.712 with only transient declines (Figure 7b).

**Figure 8:**
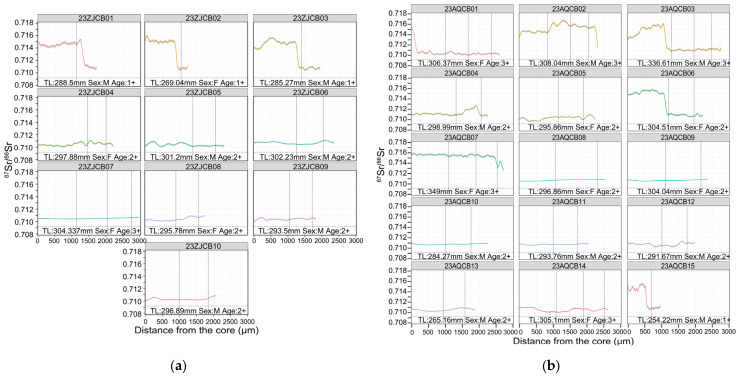
Otolith ^87^Sr/^86^Sr profiles of *Coilia brachygnathus* from the Middle–Lower Yangtze River mainstem, 2023. The position of the vertical dashed lines represents the annuli. (**a**) The specimen from Zhenjiang reach of Yangtze River; (**b**) the specimen from Anqing reach of Yangtze River.

In the MLY mainstem, Zhenjiang reach, individuals 23ZJCB01–03 had high core values (>0.714) that later declined toward 0.710, consistent with movement from Poyang Lake to the MLY mainstem. The remaining Zhenjiang fish stayed between 0.710 and 0.711 throughout (Figure 8a). In Anqing reach, individuals 23AQCB01, 02, 03, 06, 07, and 15 possessed high core values similar to Poyang Lake (>0.714), then declined to 0.710–0.711, and the trajectory of 23AQCB01 dropped rapidly, with only the inner ~50 µm retaining the high value before a swift decline, indicating movements from Poyang Lake to the MLY mainstem. In contrast, 23AQCB02 remained >0.714 until near the edge, where it finally decreased. Individual 23AQCB07 stayed elevated for most of the transect, dipped to ~0.713 near the edge, and then rose again before a second decline. Individual 23AQCB04 was stable near ~0.711 until ~1500 µm, rose to ~0.712, and then decreased again (Figure 8b).

The total length (TL, in mm), estimated age (in years, based on otolith annulus counts), and sex (F: female, M: male) for each *C. brachygnathus* specimen are presented in Figure 5, Figure 6, Figure 7 and Figure 8, with each fish’s ^87^Sr/^86^Sr profiles, providing a comprehensive view of the relationship between migration behavior and individual characteristics such as size, age, and sex within the population. Analysis of the relationship between the sex and migratory behavior revealed that among the individuals displaying clear migratory patterns (as defined by significant ^87^Sr/^86^Sr shifts beyond the location baseline), both sexes were equally represented. Specifically, we identified 10 female and 10 male individuals that exhibited distinct migratory behavior. This equal distribution suggests that migratory behavior in *C. brachygnathus* is not sex-biased within our sampled population.

Notably, otolith transect analyses revealed that in individuals 23ZJCB02–03, 23AQCB01, 23AQCB03, 23AQCB06, 23AQCB15, 23DCCB02, 23DCCB03, and 23DTCB08, pronounced habitat shifts occurred within the first annual growth increment, corresponding to the early juvenile stage (age-0 to age-1).

## 4. Discussion

This study represents the first application of otolith ^87^Sr/^86^Sr isotope analysis to elucidate habitat use patterns in *C. brachygnathus* within the MLY, revealing the species’ spatial utilization characteristics across the river–lake network. Comparative analysis of water isotope baselines and individual otolith transects indicates significant variation in ^87^Sr/^86^Sr signatures, with multiple environmental use patterns observed among individuals, demonstrating life-history complexity and plasticity that extends beyond the traditional freshwater resident hypothesis.

### 4.1. Life-History Strategies of C. brachygnathus

*C. brachygnathus* has conventionally been considered a resident species in the lakes and the mainstem of MLY basin. This differs from its closely related anadromous congener, *C. nasus*, which spends extended periods in marine or estuarine environments and exhibits stable elevated Sr/Ca ratios [18,19,20]. Although a few earlier studies reported putative estuarine life histories for isolated individuals [21], such cases may reflect rare vagrancy or artifacts of seasonal shifts in elemental concentrations that transiently elevate Sr (e.g., Sr/Ca), rather than sustained marine use. Critically, whether *C. brachygnathus* undertakes migrations within fresh water has remained unverified. In this study, the high-resolution otolith ^87^Sr/^86^Sr profiles provide direct evidence for movement between different freshwater regions, like lakes and the MLY mainstem. The edge ^87^Sr/^86^Sr ratios of most individuals matched the geochemical baseline of their capture locations, confirming their presence there prior to capture; however, departures in core and along-transect ratios reveal early-life or historical migrations (e.g., individuals 23AQCB01, 23AQCB03, and 23AQCB06). The otolith-edge ^87^Sr/^86^Sr ratios of four Shijiu individuals and two from Anqing departed from local water baselines, indicating recent movement into those reaches rather than prolonged residence.

The population exhibits multiple habitat use patterns. The first group showed stable ^87^Sr/^86^Sr profiles with fluctuations remaining within the range of their capture locations, as observed in most individuals from Shijiu Lake, Poyang Lake, Dongting Lake, and the mainstem. Minor fluctuations observed around the time of annulus formation (e.g., in individuals 23HKCB01 and 23HKCB07) may originate from seasonal variations in ambient water ^87^Sr/^86^Sr ratios, potentially driven by changes in tributary inflow and watershed weathering rates across seasons [37]. Furthermore, inter-tributary differences in ^87^Sr/^86^Sr likely generate subtle spatial variability within the lake, such as within Poyang Lake, the Xiu, Gan, Fu, and Rao Rivers carry relatively radiogenic ^87^Sr/^86^Sr with broad ranges, whereas the Xin River is less radiogenic (~0.712). As a result, waters near tributary–lake confluences can differ from the fully mixed pelagic lake, generating within-lake isotopic heterogeneity. This heterogeneity may explain the small-amplitude ^87^Sr/^86^Sr oscillations observed in some individuals. Nevertheless, the limited isotopic contrast between Shijiu Lake, the MLY mainstem, and the Dongting basin likely renders movements among these environments cryptic in otolith records, leading to underestimation of inter-habitat migration in *C. brachygnathus*.

The second group exhibited significant ^87^Sr/^86^Sr variations indicating habitat shifts. For instance, multiple individuals captured in the Anqing and Zhenjiang sections (e.g., 23AQCB01, 02, 03; 23ZJCB01, 02, 03) displayed core ^87^Sr/^86^Sr signatures of the Poyang Lake basin (>0.714), subsequently decreasing to typical MLY mainstem values (~0.710) (Figure 8a,b). This strongly suggests these individuals originated in the Poyang Lake system and migrated downstream to the MLY mainstem at some life stage. Based on the timing of isotopic inflections along otolith transects, most habitat shifts occur during subadult or adult stages, after the first annulus, when swimming capacity is fully developed. These movements are therefore best interpreted as active habitat choices rather than passive displacement. In Poyang Lake, pronounced winter drawdown and contraction of the lake area intensify density-dependent competition, likely prompting some fish to move into tributaries or exit via the outlet to the MLY mainstem, where stage and discharge fluctuate less and habitat stress is reduced, thereby improving the survival probability [45,46]. Reverse migration was also observed: one individual (23HKCB12) moved into Poyang after age-1 during spring–summer, when lake productivity favors rapid growth. This shift may reflect opportunistic exploration, but its outcome is evidently advantageous. exemplified by individual 23HKCB12 with an early life history indicating MLY mainstem (or potentially Dongting Lake) origin, later migrating to Poyang Lake (Figure 6a,b).

In contrast to these movements of subadult/adult, the pronounced early-life decline in ^87^Sr/^86^Sr for 23AQCB01 most likely reflects passive export to the MLY mainstem shortly after hatching, before the development of sustained swimming capacity [47]. *C. brachygnathus* releases pelagic eggs and typically spawns in slow moving waters or backwater bay, yet episodic currents can entrain eggs and larvae, advecting them out of spawning and nursery areas over considerable distances [14]. Similar passive drift from Poyang Lake into the mainstem has been reported for *C. nasus*.

Migration between tributaries and lake areas was also evident. Individual 23DCCB02 moved from the Xin River (0.713) to Poyang Lake, while Dongting Lake individuals (23DTCB02, 06, etc.) decreased from relatively high core values (~0.712) to lower values (~0.710), potentially indicating movement from Xiang River birthplaces to the lake area (Figure 7a,b). Conversely, Xiang River individuals (23XJCB04, 10, etc.) increasing from low to high values demonstrate movement from lake areas to tributaries. Individual 23DCCB03, primarily residing in Poyang Lake, may have temporarily entered the Xin River. These results demonstrate bidirectional migration between lakes and the MLY mainstem and between lakes and tributaries.

Complex migration patterns were observed in some individuals. Individual 23AQCB07 potentially migrated from Poyang Lake to the Xin River before moving downstream to the MLY mainstem. In other cases, however, the observed ^87^Sr/^86^Sr fluctuations cannot be fully explained by the current isotopic baseline. For instance, individual 23AQCB04 exhibited a rise in ^87^Sr/^86^Sr from 0.711 to higher than 0.712, which may indicate movement from the MLY or Dongting Lake into the Xiang River, followed by a return to the MLY mainstem near the Anqing reach, implying a cumulative displacement exceeding 1000 km. However, we acknowledge that such reconstructions are constrained by the spatial incompleteness of the water ^87^Sr/^86^Sr baseline, particularly the absence of data from smaller downstream tributaries. Consequently, observed isotopic shifts could, in some cases, reflect temporary occupation of these unsampled minor water bodies, rather than movements across major basins. This uncertainty highlights a potential source of uncertainty in the life-history interpretation of individuals with complex profiles. To reduce this ambiguity, future work should aim to systematically incorporate small tributaries and backwater channels into the high-resolution ^87^Sr/^86^Sr isoscape for the MLY to enable more definitive reconstructions of freshwater migrations.

Our findings demonstrate that migratory behavior in *C. brachygnathus* is exhibited by both sexes in equal proportions (10 females and 10 males among clear migrants), indicating the absence of strong sexual dimorphism in movement patterns. This contrasts with some fish species where migratory behavior is often sex-biased due to reproductive strategies or energetic constraints [48,49]. The equal participation of both sexes in migration suggests that the ecological drivers for movement, whether related to foraging optimization, habitat selection, or other factors, affect male and female *C. brachygnathus* similarly. This pattern may reflect the species’ life-history strategy in the dynamic Yangtze River system, where both sexes benefit from the resource opportunities provided by accessing different habitats throughout their ontogeny.

### 4.2. Biological and Ecological Implications

These findings reveal that *C. brachygnathus* exhibits a mixed life-history strategy, comprising both resident and migratory individuals within the population. These migrations are likely seasonal or staged, relating to life activities such as reproduction, feeding, or responding to environmental changes. Consequently, the species’ ecotype should be understood as a continuum of life-history strategies rather than a single resident classification.

The life-history diversity observed in *C. brachygnathus* has important ecological and evolutionary implications. This plasticity represents a key adaptive strategy to environmental heterogeneity. In the highly dynamic Yangtze Basin, connected lakes and the mainstem differ in productivity, hydrological conditions, and refuge availability [45,46]. Individual movement optimizes growth and survival probabilities, enhancing the overall population fitness. Furthermore, these results provide new perspectives on ecological differentiation between *C. brachygnathus* and its closely-related species *C. nasus*. Some genetic studies suggest that this species is a freshwater derivative of *C. nasus* [17]. Within freshwater systems, *C. brachygnathus* exhibits a flexible partial-migration strategy, with some individuals remaining within restricted ranges and others moving among tributaries, lakes, and the mainstem. This behavioral diversification likely reduces competition for spatial resources and likely generates a portfolio effect. Subpopulations with different migration behaviors respond differently to local environmental disturbances (e.g., drought, flooding, pollution), spatially and temporally dispersing the utilization of lake, mainstem, and tributary habitats. This risk-spreading buffers interannual fluctuations and enhances population stability and persistence [29].

Similar patterns are documented across many systems. In California’s Central Valley, emerging evidence shows that under altered flow regimes, maintaining life-history diversity and connectivity enhances population resilience [33,34]. Parallel results for Central Valley Chinook Salmon (*Oncorhynchus tshawytscha*) indicate that habitats once deemed marginal or non-natal can unexpectedly subsidize spawning populations, with life-history plasticity challenging conventional habitat-assessment paradigms [33,34]. In Bristol Bay, *Oncorhynchus nerka* subpopulations stabilize returns through spatially dispersed spawning across rivers and lakes [9]. Otolith ^87^Sr/^86^Sr studies from the upper Amazon document freshwater migrations spanning hundreds of kilometers in several goliath catfish (*Brachyplatystoma platynemum*) [50], paralleling the lake–river cross-system use observed here. Collectively, these findings argue for integrative conservation strategies that couple the maintenance of life-history diversity with habitat restoration and flow management to recover key ecosystem functions and metapopulation dynamics.

Historically, the MLY mainstem was laterally connected to numerous natural, shallow floodplain lakes, forming an integrated river–lake ecosystem [51,52]. Many fishes evolved river–lake migratory life-history strategies tuned to this seasonal flood regime: adults spawn in the river during high water, and early life stages are advected into connected lakes where they feed and develop. It is currently widely believed that the four major Chinese carp species, black carp (*Mylopharyngodon piceus*), grass carp (*Ctenopharyngodon idella*), silver carp (*Hypophthalmichthys molitrix*), and bighead carp (*Hypophthalmichthys nobilis*), exhibit staged migrations between the mainstem and connected lakes, spawning in the mainstem and utilizing lakes for feeding and growth [46,51]. However, current understanding is based largely on surveys of eggs, larval, and juvenile assemblages used to infer stage-specific distributions, and direct reconstructions of migratory life histories remain scarce. This study validates the feasibility of using ^87^Sr/^86^Sr to resolve life histories in freshwater systems. It offers a reliable technical approach for studying habitat use and connectivity requirements in freshwater fishes and provides quantitative evidence for understanding ecological strategies of migratory species in river–lake ecosystems.

All *C. brachygnathus* individuals in this study were captured in 2023, with the final annulus in their otoliths corresponding to early 2023. Consequently, the growth increment preceding the last annulus is interpreted as representing the 2022 life-history period. Analysis of otolith ^87^Sr/^86^Sr profiles revealed that several individuals underwent distinct habitat transitions during 2022. Notably, these movements were not only spatially diverse but also occurred at different life stages, with a significant proportion taking place during the early juvenile phase. Specifically, in 2022, individuals 23ZJCB02–03 and 23AQCB15 shifted from Poyang Lake to the Middle-Lower Yangtze (MLY) mainstem. Individual 23DCCB02 moved from the Xin River to the Poyang Lake region, while 23DCCB03 exhibited the reverse trajectory, migrating from Poyang Lake to the Xin River. Similarly, individuals 23XJCB12–14 transitioned from the Dongting Lake area to the Xiang River, whereas 23DTCB02 moved in the opposite direction, from the Xiang River into the lake region.

The habitat shifts in 2022, a year characterized by an extreme drought in the Yangtze Basin, suggests that environmental forcing may trigger dispersal responses even in young fish and demonstrates that *C. brachygnathus* exhibits significant life-history plasticity at an early juvenile stage. The fact that these movements occurred across multiple hydrological pathways (lake–river, river–lake, and within-lake systems) further underscores the species’ adaptive capacity to navigate heterogeneous freshwater landscapes under stressful conditions.

It is important to note that our sampling was conducted in 2023, following the extreme 2022 drought. While we documented a complex mosaic of resident and migratory life histories, this study provides a snapshot of the population’s state in a drought context and lacks pre-drought data for direct temporal comparison. Therefore, we cannot definitively attribute the observed patterns to the drought itself. Instead, our findings indicate that individuals such as 23DCCB13, 23AQCB03, and 23AQCB06 also undertook migrations as early as 2021, demonstrating that diverse migratory strategies were already present in the population prior to the drought. The presence of migratory individuals is consistent with the theoretical expectation that habitat fragmentation and resource scarcity caused by severe droughts can promote dispersal. However, future studies involving long-term continuous monitoring are required to establish direct causal links between specific hydrological events and shifts in life-history strategies.

### 4.3. Implications for Ecological Research and Management in the Yangtze Basin

Our findings necessitate updates to current conservation strategies. First, maintaining and restoring habitat connectivity is crucial. The results indicate that during the basin-wide extreme drought of 2022, a subset of *C. brachygnathus* shifted from floodplain lakes into the MLY mainstem or tributaries of lake, demonstrating active habitat selection and behavioral adjustment. This response underscores the population-level importance of life-history diversity. From a management perspective, these findings call for recalibration of current conservation strategies. Maintaining and restoring habitat connectivity is essential [53]. Isotopic evidence confirms that the high ^87^Sr/^86^Sr in core and low ^87^Sr/^86^Sr edge pattern observed in individuals from Anqing and Zhenjiang points to a Poyang origin and suggests movement decisions when natal habitat quality declines. The lake–river connectivity is therefore a necessary condition for mitigating climate extremes and sustaining populations under crisis conditions [54,55]. The migrated individuals in Anqing and Zhenjiang suggest Poyang Lake may be a source population for downstream areas. Future water isotope monitoring combined with mixing model analysis could quantify contributions from various lakes to mainstem populations, providing a quantitative basis for post-fishing ban resource recovery assessment [1,33].

Furthermore, integrating otolith ^87^Sr/^86^Sr with complementary stable isotopes (δ^13^C, δ^15^N) and microstructure-derived daily age, together with basin-wide water isotope monitoring and mixing-model analyses will enable dynamic tracking at the individual, population, and ecosystem levels [31,56]. This framework can quantify lake-specific contributions to mainstem assemblages, provide quantitative baselines for evaluating resource recovery after the fishing ban, and generate biological early-warning indicators by detecting interannual shifts in the proportions of life-history types [1,33]. These indicators can be used to assess the effects of flow regulation, lake degradation, and restoration interventions. In addition, the otolith isotope “natal-to-adult matching” approach is transferable to other important species to identify nurseries and prioritize habitat restoration.

## 5. Conclusions

In conclusion, otolith ^87^Sr/^86^Sr records show that under an extreme-drought climate context, *C. brachygnathus* in the MLY river–lake network expressed multiple life-history strategies, including local residency and stage-specific active migrations. This finding challenges the traditional view of freshwater residency and demonstrates the power of Sr-isotope ecology to resolve habitat use in low-salinity systems. The results provide new scientific support for managing lake–river connectivity, rebuilding fisheries, and sustaining ecosystem functions, and they offer quantitative tools and conceptual guidance for basin-scale ecological operations and habitat protection.

## Figures and Tables

**Table 1:** Sampling locations of *Coilia brachygnathus* and sample characteristics. *N*: number; TL: total length (mean ± SD), SL: standard length (mean ± SD), W: body weight (mean ± SD).

Region	Location	Number	Total Length	Standard Length	Body Weight
Poyang Lake	Duchang	15	305.65 ± 12.21	286.73 ± 11.83	74.09 ± 13.94
	Hukou	15	292.58 ± 33.19	272.00 ± 31.68	69.30 ± 23.99
Dongting Lake	Yueyang	15	294.09 ± 33.41	273.52 ± 31.60	75.97 ± 28.55
	Xiangyin	14	307.79 ± 11.51	289.42 ± 11.62	83.60 ± 12.60
Shijiu Lake	Shijiu Lake	23	280.81 ± 12.69	261.79 ± 12.63	58.23 ± 13.59
Yangtze River	Anqing	15	299.63 ± 23.30	281.38 ± 21.73	81.96 ± 20.27
	Zhenjiang	10	293.46 ± 10.44	270.29 ± 13.27	65.08 ± 10.67

**Table 2:** Summary of water ^87^Sr/^86^Sr baselines for the major locations in the study area. The lower and upper represent the mean minus and plus one standard deviation (mean ± SD), respectively.

Region	Location	Number	Mean	Standard Deviation	Lower	Upper
Dongting Basin	Dongting	4	0.71115	0.00056	0.71058	0.71171
	Xiang River	2	0.71255	0.00033	0.71223	0.71288
	Yuan River	3	0.71167	0.00019	0.71147	0.71186
	Zi River	2	0.71178	0.00030	0.71147	0.71208
Shijiu Lake	Shijiu Lake	2	0.71010	0.00108	0.70902	0.71118
Poyang Basin	Poyang	4	0.71445	0.00055	0.71390	0.71499
	Gan River	8	0.71942	0.00261	0.71681	0.72203
Han River	Han River	8	0.71202	0.00079	0.71123	0.71281
Yangtze River	Yangtze River	22	0.71057	0.00026	0.71031	0.71082

## Data Availability

Data for this research article are available from the corresponding authors by reasonable request.

## Abbreviations

The following abbreviations are used in this manuscript:

MLY	the middle and lower reaches of the Yangtze River
GAM	Generalized Additive Models (GAM)

## Appendix A

**Table A1:** Detailed information for each individual water sample.

Sample Name	Latitude	Longitude	87Sr/86 Sr	Sampling Site	Collection Date	References
CJ-59	29.377	113.079	0.71190	Dongting Lake	2006.08	Chetelat et al. [38]
DTH	29.434	113.118	0.71090	Dongting Lake	2011.08	Luo et al. [37]
Dongting	29.305	113.049	0.71058	Dongting Lake	2023.08	This study
Dongting	29.304	113.049	0.71121	Dongting Lake	2024.03	This study
CJ-31	28.906	111.486	0.71189	Yuan River	2006.08	Chetelat et al. [38]
YJ	28.968	111.707	0.71157	Yuan River	2011.08	Luo et al. [37]
YJ	29.096	111.904	0.71154	Yuan River	2011.08	Luo et al. [37]
CJ-32	28.599	112.363	0.71156	Zi River	2006.08	Chetelat et al. [38]
ZJ	28.644	112.382	0.71199	Zi River	2011.08	Luo et al. [37]
SZ	30.24	111.85	0.71010	Songzi River	2011.08	Luo et al. [37]
CJ-33	28.123	112.947	0.71278	Xiang River	2006.08	Chetelat et al. [38]
XJ	28.255	113.087	0.71232	Xiang River	2011.08	Luo et al. [37]
CJ-43	29.645	116.204	0.71514	Poyang Lake	2006.08	Chetelat et al. [38]
PYH	29.861	116.351	0.71441	Poyang Lake	2011.08	Luo et al. [37]
Piaotou	29.196	116.191	0.71445	Poyang Lake	2023.08	This study
Duchang	29.206	116.268	0.71380	Poyang Lake	2024.01	This study
FH	28.345	116.076	0.71679	Fu River	2023.08	This study
RH	29.024	116.629	0.713862	Rao River	2023.08	This study
XJ	28.725	116.422	0.71262	Xin River	2023.08	This study
XS-01	29.061	115.828	0.72062	Xiu River	2023.08	This study
CJ-34	28.67	115.868	0.71534	Gan River	2006.08	Chetelat et al. [38]
GJ10	26.619	114.756	0.72030	Gan River	2007.11	Wang et al. [41]
GJ09	26.555	114.671	0.72350	Gan River	2007.11	Wang et al. [41]
GJ13	27.151	115.042	0.72180	Gan River	2007.11	Wang et al. [41]
GJ15	27.362	115.108	0.72040	Gan River	2007.11	Wang et al. [41]
GJ18	27.769	115.384	0.71850	Gan River	2007.11	Wang et al. [41]
GJ20	28.16	115.659	0.71830	Gan River	2007.11	Wang et al. [41]
GJ22	28.565	115.808	0.71720	Gan River	2007.11	Wang et al. [41]
CJ-47	30.597	114.081	0.71151	Han River	2006.08	Chetelat et al. [38]
HJ-49	32.469	111.586	0.71258	Han River	2007.09	Xu et al. [40]
HJ-50	32.339	111.622	0.71307	Han River	2007.09	Xu et al. [40]
HJ-56	31.148	112.545	0.71143	Han River	2007.09	Xu et al. [40]
HJ-57	30.726	112.607	0.71148	Han River	2007.09	Xu et al. [40]
HJ-60	30.389	113.479	0.71139	Han River	2007.09	Xu et al. [40]
HJ-62	30.564	114.284	0.71150	Han River	2007.09	Xu et al. [40]
HJ-54	32.051	112.198	0.71320	Han River	2007.09	Xu et al. [40]
SJH_Guxihe	31.529	118.663	0.709335	Shijiu Lake	2024.01	This study
SJH_Guxihe	31.555	118.466	0.710858	Shijiu Lake	2023.08	This study
CJ03	31.964	120.795	0.71077	Yangtze River	1997.10	Wang et al. [41]
CJ04	32.034	120.526	0.71071	Yangtze River	1997.10	Wang et al. [41]
CJ05	31.949	120.089	0.71073	Yangtze River	1997.10	Wang et al. [41]
CJ06	32.304	119.79	0.71072	Yangtze River	1997.10	Wang et al. [41]
CJ07	32.178	118.878	0.71074	Yangtze River	1997.10	Wang et al. [41]
CJ08	31.623	118.417	0.71065	Yangtze River	1997.10	Wang et al. [41]
CJ09	31.225	118.021	0.71074	Yangtze River	1997.10	Wang et al. [41]
CJ11	30.442	116.955	0.71067	Yangtze River	1997.10	Wang et al. [41]
CJ12	29.796	116.252	0.71066	Yangtze River	1997.10	Wang et al. [41]
CJ13	29.758	115.861	0.71054	Yangtze River	1997.10	Wang et al. [41]
CJ14	30.436	114.841	0.71058	Yangtze River	1997.10	Wang et al. [41]
CJ16	30.033	113.916	0.71055	Yangtze River	1997.10	Wang et al. [41]
CJ17	29.757	113.451	0.71045	Yangtze River	1997.10	Wang et al. [41]
CJ18	29.772	112.896	0.71029	Yangtze River	1997.10	Wang et al. [41]
CJ19	30.297	112.103	0.70997	Yangtze River	1997.10	Wang et al. [41]
CJ20	30.278	111.547	0.71003	Yangtze River	1997.10	Wang et al. [41]
CJ21	30.825	111.023	0.71031	Yangtze River	1997.10	Wang et al. [41]
SX5	30.954	110.754	0.71054	Yangtze River	2006.08	Chetelat et al. [38]
CJ-42	31.98	120.679	0.71100	Yangtze River	2006.08	Chetelat et al. [38]
CJ-44	29.903	116.542	0.71099	Yangtze River	2006.08	Chetelat et al. [38]
Anqing	30.457	116.946	0.71035	Yangtze River	2023.08	This study
ZJ	32.263	119.474	0.71057	Yangtze River	2023.08	This study
